# Polyposis syndromes– what to do when genotyping seems not informative

**DOI:** 10.1186/1897-4287-10-S2-A2

**Published:** 2012-04-12

**Authors:** F Macrae

**Affiliations:** 1 Head, Colorectal Medicine and Genetics, The Royal Melbourne Hospital, Australia

## Background

In polyposis, the type and numbers of polyps, the pattern of inheritance, and any extraintestinal features are all important in strategic planning of the DNA mutational approach. But where should we go when no mutation is uncovered? This paper outlines an approach we have adopted at RMH.

## Multiple adenomatous polyps

As a routine, we check to see if both sequencing and MLPA has been complete for APC, and full sequencing for MYH (though the return outside the three common mutations is low in our ethnic mix). On at least one occasion, a change in the MLPA kit by Holland Inc lead to identification of an important deletion as the probes changed in the MLPA kit. So recollecting blood to run on a new kit can be informative. RNA studies may also identify expression perturbations leading to closer scrutiny of the DNA. We then look for translocations by FISH analysis. If all this is negative, we move the patient to a research setting; Justine Marum is well into a PhD evaluating the role of *AXIN* mutations in this group of patients. The findings to date are being confirmed with other approaches to establishing pathogenicity of the *AXIN* variants identified. This work is being done also in collaboration with Dr Marie Faux at the Ludwig Institute of Cancer Research and Prof Rodney Scott at University of Newcastle.

Clinically, patients with multiple adenomas even without a germline mutation identified, are at increased risk of colorectal cancer; indeed, multiplicity of adenomas is the highest risk factor of subsequent colorectal cancer identifiable. The new NHMRC guidelines out for public comment recommend follow up of patients: *As multiplicity of adenomas is a strong determinant of risk of metachronous advanced and non advanced neoplasia*, *follow up should be at twelve months for those with five or more adenomas*, *and sooner in those with ten or more adenomas.*

The risk of colorectal cancer to relatives of patients with multiple adenomas is related to the number of adenomas in the proband – outside FAP and MYH. Therefore, as distinct from patients with small numbers of adenomas, relatives of patients with larger numbers (10+) should be offered colonoscopy at an age relative to the age of presentation of the proband. The interval is not well defined but I would suggest 5 yearly.

## Hyperplastic polyposis

HPS has no defined genetic basis as yet. These patients are at high risk of colorectal cancer, either through the serrated pathway or through their concurrent adenomas. Two yearly colonoscopy is advised. Although the mode of inheritance is also poorly defined, it is thought it may be recessive. Therefore siblings are offered surveillance, though usually not expected to be found with HPS themselves. There is however, a high risk of colorectal cancer in first degree relatives (siblings or parents) so colonoscopy should be offered to these relatives 5 yearly.

## Low level mosaicism, gonadal mosaicism, somatic mosaicism

Somatic mosaicism is now well described in FAP, with respect to the APC gene; cases of multiple adenomatous polyposis may therefore be found with no germline (lymphocytic) APC mutation. We have been searching for the same APC mutation in multiple polyps, and in background mucosa, in these patients without definitive progress, but others have reported this phenomenon. Some of this can be traced to low level mosaicism including even in the germline (lymphocytes) but not detected with standard approaches to APC mutational analysis. We have also been interested to seek evidence of gonadal mosaicism in the parents of apparent *de novo* cases, and are still pursuing this possibility. This requires predictive DNA testing of all siblings of apparent *de novo* cases, and their parents. Gonadal mosaicism in FAP has been reported in Utah. Recently we have had occasion to consider whether mosaicism may involve both the gonads and the colon – through a request to colonoscope parents who tested negative for an apparent *de novo* APC mutation in their only child. This has lead to a range of opinions and experiences described from colleagues around the world which will be of interest to the FCC counsellors.

